# Book Review: Perception of Pixelated Images

**DOI:** 10.3389/fpsyg.2016.01151

**Published:** 2016-08-12

**Authors:** Valérie Goffaux

**Affiliations:** ^1^Psychological Sciences Research Institute, Université Catholique de LouvainLouvain-la-Neuve, Belgium; ^2^Institute of Neuroscience, Université Catholique de LouvainLouvain-la-Neuve, Belgium; ^3^Cognitive Neuroscience Department, Maastricht UniversityMaastricht, Netherlands

**Keywords:** quantization, pixelation, spatial frequency, recognition, digitization

Despite the fact that we feel immersed in a rich and continuous flow of visual sensations, our visual system samples only a small fraction of the luminance variations present in the environment. Such sparse sampling inevitably comes along with a loss of information. And this is advantageous since it decreases the computational and metabolic needs of the system to e.g., generate, classify, and store images. But sampling must be smartly calibrated so that critical cues are not lost. This seems to be the case for the perception of major visual categories, such as faces and letters, which has been found to rely on a restricted but optimized range of spatial resolutions, also called spatial frequencies (SF; Gold et al., 1999; Nasanen, 1999; Majaj et al., 2002).

Initial works addressing the SF dependency of human perception manipulated image spatial resolution by means of quantization, also called pixelation. In his recent book, Talis Bachmann reviews how this method contributed to a better understanding of human vision. Quantization consists in dividing an image into equally sized squares, and filling each square with its averaged luminance value (Figures 1A,B). This image process acts like a low-pass SF filter since it maintains the coarse structure of the original picture (i.e., its low SF) but removes its finer details (i.e., its high SF). But quantization also produces a spurious block structure, which adds “alien” high SF to the image.

**Figure 1:**
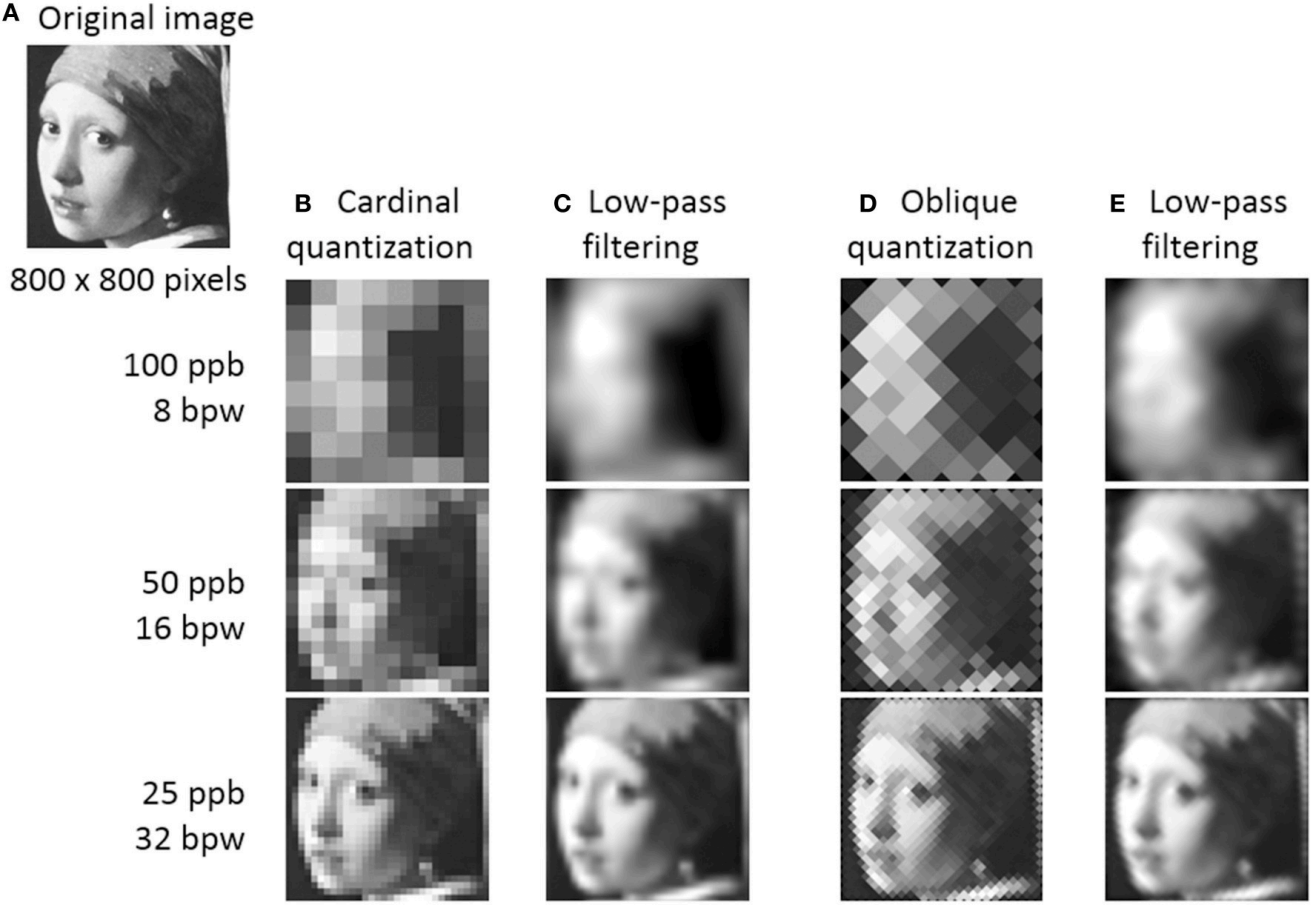
**(A)** Grayscale detail (800 by 800 pixels) of “Girl with a Pearl Earring,” oil painting by Johannes Vermeer. **(B)** The image has been quantized at different spatial scales, by averaging luminance over square areas of different sizes. Spatial scale of quantized images can be expressed in two ways: block size (i.e., pixels per block or ppb) or the number of blocks per image width (bpw) or height. The latter measure divided by two can be taken to approximate the SF range available in the quantized image. To be recognized, a quantized image of a face needs to contain at least 16 blocks (e.g., approximately 8 cycles per image; e.g., Bachmann, 1991). The sharp edges of the spurious blocks that occupies high SF directly adjacent to the low SF range of portrait (from 4 cycles per image on for an 8-block quantized image) largely disrupt image recognizability. **(C)** When block edges are attenuated by low-pass filtering, the recognition of the quantized image improves (Harmon and Julesz, 1973). (**D,E**) Obliquely-quantized images seem to provide cues that do not fully overlap with those carried by cardinally-quantized images **(B,C)**. Using different block structure orientations may yield new insights on how quantization affects shape processing.

The quantization adventure started with the work published by Harmon and Julesz (1973). The authors quantized the iconic portrait of President Abraham Lincoln and found that portrait recognizability decreased as block size increased (Figures 1A,B). Interestingly, the recognition of the quantized portrait recovered to some extent when block edges were attenuated by low-pass SF filtering (Figure 1C). Harmon and Julesz (1973) interpreted this observation as reflecting “critical band masking,” namely that the high SF of the block structure interfere with (or mask) the low SF carrying portrait information. Such masking was proposed to emerge at primary visual stages of SF extraction, before the integration of visual input into a shape.

Later Morrone et al. (1983) challenged the early “critical band masking” interpretation by reporting a seemingly paradoxical finding: portrait recognition improves when high SF random noise is added to the quantized image. If the disruptive effects of quantization on perception were due to inter-SF competition, increasing the power of high SF by adding noise should even more interfere with the recognition of the low SF portrait. That portrait recognition improves when block shape is destroyed by noise instead suggests that the difficulty of recognizing quantized images is due to a competition between the integration of block and portrait *shapes*, at a higher visual processing stage than the early SF extraction stage (Bachmann and Kahusk, 1997; see also Caelli and Yuzyk, 1985). Besides the disruptive effect of the high SF block edges on perception, quantization was also reported to distort the second-order properties of the low SF image content (Caelli and Yuzyk, 1985; Bachmann and Kahusk, 1997; Morgan and Watt, 1997; Morrone and Burr, 1997). Although quantization was initially used to investigate the primary SF dependencies of human vision, this evidence shows that it also drastically distorts the higher-level (shape) properties of the image.

Actually, quantization also affects the orientation content of the image. Considering that (1) the visual system preferentially responds to cardinally-oriented edges (at least for meaningless shapes; Furmanski and Engel, 2000) and that (2) distinct orientation ranges are optimal for the perception of core categories such as faces and scenes (Hansen et al., 2003; Dakin and Watt, 2009; Goffaux and Dakin, 2010; Pachai et al., 2013), it is plausible that the standard cardinal orientation of block averaging influenced quantization evidence in peculiar and complex ways. Using a different quantization structure (Figures 1D,E) may yield new insights on the shape-related mechanisms involved when dealing with quantized images.

Because quantized image perception actually reflects complex and still elusive interactions between the integration of block and e.g., portrait *shapes*, interpreting perceptual findings derived from this technique proves difficult (Costen et al., 1994; Morrison and Schyns, 2001). Therefore, most researchers investigating the optimal SF range for human vision abandoned quantization in favor of Fourier-filtering procedures. As a consequence, the empirical literature related to quantization is relatively limited. The present book describes in detail this confined literature, without providing innovative arguments that would potentially make the reader reconsider the contribution of this technique to the field of vision science. Bachmann defends quantization as a more valid means to manipulate visual perception than SF filtering due to its more disruptive effect on shape integration. However, the elusiveness of quantization effects on shape processing undermines this statement.

Research on quantization may be more illuminating with regards to digital sampling. These last decades the amount of image data on the internet has exploded (e.g., Deng et al., 2009), and our everyday visual diet has dramatically changed to become increasingly digital. Analogously to images captured by our visual system, the apparently smooth and rich digital images result from a sampling operation that break luminance gradients of the captured scene into discrete units called pixels. As Bachmann states, both visual and digital sampling are bound to the spatial resolution issue, i.e., how fine-grained an image should be to allow for recognition by man and machine. Quantization evidence has the potential to inform on the spatial resolution necessary for an economic storage of digital images, the optimal image classification by computer algorithms, and ultimately the development of efficient artificial intelligent devices. The book casts some light on these potential and more warranted contributions of quantization research.

## Author contributions

The author confirms being the sole contributor of this work and approved it for publication.

## Funding

The author is supported by the Belgian National Foundation for Scientific Research (F.R.S.-F.N.R.S.).

### Conflict of interest statement

The authors declare that the research was conducted in the absence of any commercial or financial relationships that could be construed as a potential conflict of interest.
